# Contribution of Eat1 and Other Alcohol Acyltransferases to Ester Production in *Saccharomyces cerevisiae*

**DOI:** 10.3389/fmicb.2018.03202

**Published:** 2018-12-21

**Authors:** Aleksander J. Kruis, Brigida Gallone, Timo Jonker, Astrid E. Mars, Irma M. H. van Rijswijck, Judith C. M. Wolkers–Rooijackers, Eddy J. Smid, Jan Steensels, Kevin J. Verstrepen, Servé W. M. Kengen, John van der Oost, Ruud A. Weusthuis

**Affiliations:** ^1^Laboratory of Microbiology, Wageningen University and Research, Wageningen, Netherlands; ^2^Bioprocess Engineering, Wageningen University and Research, Wageningen, Netherlands; ^3^Department of Plant Biotechnology and Bioinformatics, Ghent University, Ghent, Belgium; ^4^VIB-UGent Center for Plant Systems Biology, Ghent, Belgium; ^5^VIB-KU Leuven Center for Microbiology, Leuven, Belgium; ^6^Laboratory of Genetics and Genomics, Centre of Microbial and Plant Genetics, Department of M2S, KU Leuven, Leuven, Belgium; ^7^Leuven Institute for Beer Research (LIBR), Leuven, Belgium; ^8^Biobased Products, Wageningen University and Research, Wageningen, Netherlands; ^9^Laboratory of Food Microbiology, Wageningen University and Research, Wageningen, Netherlands

**Keywords:** Eat1p, alcohol acyltransferase, AAT, ester, yeast, *Saccharomyces cerevisiae*, wine

## Abstract

Esters are essential for the flavor and aroma of fermented products, and are mainly produced by alcohol acyl transferases (AATs). A recently discovered AAT family named Eat (Ethanol acetyltransferase) contributes to ethyl acetate synthesis in yeast. However, its effect on the synthesis of other esters is unknown. In this study, the role of the Eat family in ester synthesis was compared to that of other *Saccharomyces cerevisiae* AATs (Atf1p, Atf2p, Eht1p, and Eeb1p) *in silico* and *in vivo*. A genomic study in a collection of industrial *S. cerevisiae* strains showed that variation of the primary sequence of the AATs did not correlate with ester production. Fifteen members of the *EAT* family from nine yeast species were overexpressed in *S. cerevisiae* CEN.PK2-1D and were able to increase the production of acetate and propanoate esters. The role of Eat1p was then studied in more detail in *S. cerevisiae* CEN.PK2-1D by deleting *EAT1* in various combinations with other known *S. cerevisiae* AATs. Between 6 and 11 esters were produced under three cultivation conditions. Contrary to our expectations, a strain where all known AATs were disrupted could still produce, e.g., ethyl acetate and isoamyl acetate. This study has expanded our understanding of ester synthesis in yeast but also showed that some unknown ester-producing mechanisms still exist.

## Introduction

Volatile esters are produced by yeasts during fermentation and are key contributors to the flavor of fermented food products (Verstrepen et al., 2003a; Swiegers et al., 2005). Esters are present at concentrations around the human detection limit. Consequently, even small changes in ester concentrations can lead to profound changes in the beer and wine bouquets (Saerens et al., 2010). Esters generally add a fresh fruity aroma to beverages. However, in excessive amounts they can also lead to undesirable off-flavors (Liu et al., 2004). A well-balanced volatile ester profile is therefore key for palatable fermented products. The amounts and variety of esters produced depends highly on the strain of *S. cerevisiae* used for fermentation (Gallone et al., 2016). The main contributors to product flavor are acetate esters, including ethyl acetate (sweet aroma), isoamyl acetate (banana) and phenylethyl acetate (rose, flowery aroma) (Verstrepen et al., 2003b; Dzialo et al., 2017). The second major group of esters are medium chain fatty acid (MCFA) ethyl esters, like ethyl octanoate (sour apple) and ethyl hexanoate (apple, anise) (Knight et al., 2014).

Esters in *Saccharomyces cerevisiae* are produced by alcohol acyltransferases (AATs), which couple an acyl-CoA with an alcohol, producing esters and free CoA. MCFA ethyl esters are produced by the paralog pair Eht1p and Eeb1p, which couple an MCFA-CoA with ethanol. A Δ*eeb1* strain produced less ethyl butanoate, ethyl hexanoate, ethyl octanoate and ethyl decanoate. The deletion of *EHT1* only reduced ethyl hexanoate and ethyl octanoate levels. Overexpression of *EHT1* or *EEB1* did not increase ester production (Saerens et al., 2006). Acetate esters in *S. cerevisiae* are produced by Atf1p and its paralog Atf2p (Fujii et al., 1996; Nagasawa et al., 1998). Overexpression of *ATF1* and *ATF2* resulted in increased acetate ester production (Verstrepen et al., 2003b; Lilly et al., 2006). A Δ*atf1*Δ*atf2* deletion strain produced approximately 50% less ethyl acetate and 80% less isoamyl acetate, as well as significantly lower amounts of other acetate esters (Verstrepen et al., 2003b). However, the production of acetate esters was not entirely abolished. MCFA ethyl esters were not significantly affected by the deletion of *ATF1* and *ATF2*.

Recently, a new family of ethyl acetate producing AATs was discovered in yeasts which was named Eat (Kruis et al., 2017). These AATs are the main enzymes responsible for bulk ethyl acetate production in yeasts such as *Wickerhamomyces anomalus, Kluyveromyces marxianus*, and *Cyberlindnera fabianii*. Two putative homologs of *EAT* were identified in *S. cerevisiae*, *YGR015C* (now renamed *EAT1*) and *YGR031W*. The latter was named *IMO32* in a previous study (Vögtle et al., 2011). We have previously shown that overexpression of the *S. cerevisiae EAT1* increased ethyl acetate production in *S. cerevisiae* (Kruis et al., 2017). These results were confirmed in a study where *EAT1* was identified as a source of ethyl acetate synthesis in *S. cerevisiae* Δ*atf1* (Holt et al., 2018).

Previous studies showed that the activities of Atf1p, Atf2p, Eht1p, and Eeb1p did not account for all the ester production observed in *S. cerevisiae* (Dzialo et al., 2017). When *EAT1* was deleted in *S. cerevisiae*, ethyl acetate production was reduced by approximately 50% (Kruis et al., 2017). However, the effect of Eat1p on the production of esters other than ethyl acetate, and the effect of the combined deletions of all ester-forming AATs have not been reported yet.

This study focused on the effect of Eat1p and other AATs on ester production in *S. cerevisiae*. We performed a genomic analysis of all six AATs in a collection of industrial *S. cerevisiae* strains with known ester production profiles. We then studied the ester-forming capacities of 15 members of the *EAT* family by overexpressing them in *S. cerevisiae*. Next, we determined the contribution of *ATF1, ATF2, EHT1, EEB1, IMO32*, and *EAT1* on ester production in *S. cerevisiae* using a series of overexpression and deletion strains, including a strain lacking all six AAT-encoding genes.

## Materials and Methods

### Strain and Plasmid Construction

Strains and plasmids used and created in this study are listed in Tables 1, 2, respectively. Plasmids p414-TEF1p-Cas9-CYC1t and p426-SNR52p-gRNA.CAN1.Y-SUP4t were gifts from George Church (Addgene plasmids #43802 and #43803, respectively). The plasmid pROS13 was a gift from A. J. A. van Maris (Euroscarf plasmid #P30790). Plasmid p426-*ATF1* and p426-*EAT1* were constructed by introducing the appropriate gRNA sequence into the plasmid through PCR. A 5′ phosphoryl group was introduced on the 5′ ends of the PCR product through 5′ phosphorylated primers. The PCR product was then purified using the DNA Clean & Concentrator^TM^-5 (Zymo research) and ligated with T4 ligase (NEB) according to the manufacturer’s protocol. Plasmid p426-*ATF2* was constructed from a PCR amplified p426 backbone (Q5, NEB) and a synthetic gBlock (IDT), containing the *ATF2* gRNA sequence and 50 bp homologous regions overlapping the linear plasmid backbone. The two fragments were assembled using Gibson assembly^®^ (NEB), following the manufacturer’s instructions. pROS13-*EHT1* and pROS13-*EEB1* were constructed by introducing the appropriate gRNAs as described previously (Mans et al., 2015). gRNAs were designed with the help of ChopChop (Labun et al., 2016). Plasmids, ligations and Gibson assemblies were routinely transformed to NEB^®^ 5-alpha chemically competent cells (NEB) according to the supplier protocol. Correct plasmid construction was confirmed by sequencing (GATC, Macrogen). *S. cerevisiae* plasmid transformations were performed as described previously (Gietz and Woods, 2002). Gene disruptions in *S. cerevisiae* CEN.PK2-1D (p414-TEF1p-Cas9-CYC1t) were performed by co-transformation with the appropriate p426 or pROS13 plasmid, together with a synthetic dsDNA repair fragment (IDT), as described previously (DiCarlo et al., 2013). The 140 bp repair fragment consisted of 70 bp homology directly upstream and downstream of the start and stop codon. Successful disruptions were confirmed by PCR, using genomic DNA as template (Lõoke et al., 2011). Plasmids derived from p426-SNR52p-gRNA.CAN1.Y-SUP4t were cured by growing the strains in the presence of 1 mg/mL 5-fluoroorotic acid (Boeke et al., 1984). Plasmids derived from pROS13 were cured by overnight growth without antibiotics and subsequent streaking on selective and non-selective plates. Colonies unable to grow on selective plates were deemed cured and used for the next round of gene disruption. Plasmid p414-TEF1p-Cas9-CYC1t was cured by counter-selection with 0.5 mg/ml 5-fluoroanthranilic acid (Toyn et al., 2000). Cured strains were used for physiological characterization.

**Table 1:** Strains used and created during this study.

Strain	Origin
*Escherichia coli*	
NEB5-α	NEB
*Saccharomyces cerevisiae*	
CEN.PK2 1-D	Entian and Kötter, 2007
CEN.PK2 1-D Δ*atf1*	This study
CEN.PK2 1-D Δ*atf2*	This study
CEN.PK2 1-D Δe*ht1*	This study
CEN.PK2 1-D Δe*eb1*	This study
CEN.PK2 1-D Δ*eat1*	This study
CEN.PK2 1-D Δ*imo32*	This study
CEN.PK2 1-D Δ*atf1*Δ*atf2*	This study
CEN.PK2 1-D Δ*eeb1*Δ*eht1*	This study
CEN.PK2 1-D Δ*atf1*Δ*atf2*Δ*eat1*	This study
CEN.PK2 1-D Δ*eat1*Δ*eeb1*Δ*eht1*	This study
CEN.PK2 1-D Δ*atf1*Δ*atf2*Δ*eeb1*Δ*eht1*	This study
CEN.PK2 1-D Δ*atf1*Δ*atf2*Δ*eeb1*Δ*eht1*Δ*imo32*	This study
CEN.PK2 1-D Δ*atf1*Δ*atf2*Δ*eht1*Δ*eeb1*Δ*eat1*	This study
CEN.PK2 1-D Δ*atf1*Δ*atf2*Δ*eht1*Δ*eeb1*Δ*eat1*Δ*imo32*	This study


**Table 2:** Plasmids used and created in this study.

Construct	Characteristics	Origin
p414-TEF1p-Cas9-CYC1t	Plasmid harboring SpyCas9	DiCarlo et al., 2013
p426-SNR52p-gRNA.CAN1.Y-SUP4t	Plasmid expressing the sgRNA	DiCarlo et al., 2013
p426-*ATF1*	p426-SNR52p-gRNA.CAN1.Y-SUP4t expressing the *ATF1* targeting gRNA	This study
p426-*ATF2*	p426-SNR52p-gRNA.CAN1.Y-SUP4t expressing the *ATF2* targeting gRNA	This study
p426-*EAT1*	p426-SNR52p-gRNA.CAN1.Y-SUP4t expressing the *EAT1* targeting gRNA	This study
p426-*IMO32*	p426-SNR52p-gRNA.CAN1.Y-SUP4t expressing the *IMO32* targeting gRNA	This study
pROS13	Dual gRNA expression plasmid	Mans et al., 2015
pRos13-*EHT1*	pROS12 expressing *EHT1* targeting gRNA	This study
pRos13-*EEB1*	pROS12 expressing *EEB1* targeting gRNA	This study
pCUP1	pYES2 (Invitrogen) where the GAL1 promoter was replaced with the CUP1 promoter	Kruis et al., 2017
pCUP1: Wan Eat1	Expression of *Wickerhamomyces anomalus* DSM 6766 *EAT1*	Kruis et al., 2017
pCUP1: Wan Eat2	Expression of *Wickerhamomyces anomalus* DSM 6766 *EAT2*	Kruis et al., 2017
pCUP1: Wci Eat1	Expression of *Wickerhamomyces ciferrii* CBS 111 *EAT1*	Kruis et al., 2017
pCUP1: Wci Eat2	Expression of *Wickerhamomyces ciferrii* CBS 111 *EAT2*	Kruis et al., 2017
pCUP1: Kma Eat1	Expression of *Kluyveromyces marxianus* DSM 5422 *EAT1*	Kruis et al., 2017
pCUP1: Kla Eat1	Expression of *Kluyveromyces lactis* CBS 2359 *EAT1*	Kruis et al., 2017
pCUP1: Cja Eat1	Expression of *Cyberlindnera jadinii* DSM 2361 *EAT1*	Kruis et al., 2017
pCUP1: Cja Eat2	Expression of *Cyberlindnera jadinii* DSM 2361 *EAT2*	Kruis et al., 2017
pCUP1: Cfa Eat1	Expression of *Cyberlindnera fabianii* CBS 5640 *EAT1*	Kruis et al., 2017
pCUP1: Cfa Eat2	Expression of *Cyberlindnera fabianii* CBS 5640 *EAT2*	Kruis et al., 2017
pCUP1: Huv Eat1	Expression of *Hanseniaspora uvarum* CECT 11105 *EAT1*	Kruis et al., 2017
pCUP1: Huv Eat2	Expression of *Hanseniaspora uvarum* CECT 11105 *EAT2*	Kruis et al., 2017
pCUP1: Ecy Eat1	Expression of *Eremothecium cymbalarie* CBS 270.75 *EAT1*	Kruis et al., 2017
pCUP1: Sce Eat1	Expression of *S. cerevisiae* NCYC 2629 *EAT1*	Kruis et al., 2017
pCUP1: Sce Imo32	Expression of *S. cerevisiae* NCYC 2629 IMO32 (*EAT2* homolog)	Kruis et al., 2017


### Cultivation Conditions

*Escherichia coli* and *S. cerevisiae* cultures were routinely cultivated in LB (10 g/L tryptone, 5 g/L yeast extract, 10 g/L NaCl) and YPD medium (20 g/L glucose, 10 g/L yeast extract, 20 g/L peptone), respectively. 15 g/L bacteriological agar was added to make plates. Media were supplemented with 50 μg/mL ampicillin and 200 μg/mL geneticin when appropriate. *E. coli* and *S. cerevisiae* were cultivated at 37 and 30°C, respectively, unless stated otherwise. *S. cerevisiae* gene disruptions were performed in YNB-HL medium [5 g/L glucose, 6.7 g/L Yeast Nitrogen Base, vitamins and trace elements (Verduyn et al., 1992), 125 mg/L histidine-HCl and 500 mg/L leucine]. 75 mg/L tryptophan and 150 mg/L uracil were added to YNB-HL as needed.

*Saccharomyces cerevisiae* strains harboring pCUP1 derived plasmids were characterized in YSg medium (20 g/L glucose, 6.7 g/L Yeast Nitrogen Base without amino acids, 1.92 g/L medium supplements without uracil). Single colonies were picked into 10 mL YSg and incubated overnight while shaking at 150 rpm. The following day, 100 μL of the preculture was transferred to 10 mL fresh YSg in a 50 mL Greiner tube while shaking at 150 rpm. 1 mM CuSO_4_ was added for gene induction. Cultures were sampled after 24 h. Samples were frozen at -20°C until analysis. Experiments were performed as biological duplicates.

*Saccharomyces cerevisiae* gene disruption strains were assayed in YPD-80 (YPD medium with 80 g/L glucose) and YSg-ura (YSg medium supplemented with 150 mg/L uracil). Single colonies were picked into 10 mL medium and incubated overnight while shaking at 150 rpm. The following day, 100 μL of the preculture was transferred to 10 mL fresh medium in a 50 mL Greiner tube while shaking at 150 rpm for 24 h. 2 mL sample was transferred to a 10 mL glass vial, sealed, and frozen at -20°C until analysis. Experiments were performed as biological duplicates. Samples were analyzed as technical duplicates.

### White Grape Juice Fermentation

White grape juice was prepared by diluting the Arsegan^®^ white grape juice concentrate five times. 1 g/L yeast extract was added to the medium which was filter sterilized. Subsequent HPLC analysis revealed that 58.8 g/L glucose and 53.3 g/L fructose were initially present in the medium. Precultures were grown in 10 mL medium in a 50 mL Greiner tube while shaking overnight at 150 rpm. The next day, 2 mL of the preculture was transferred to 50 mL fresh medium in a 100 mL Schott bottle. The bottles were closed with a rubber stopper and a water lock. Cultures were cultivated statically at 20°C for 7 days. 2 mL sample was transferred to a 10 mL glass vial, sealed, and frozen at -20°C until analysis. Experiments were performed as biological duplicates. Samples were analyzed as technical duplicates.

### Analytical

OD_600_ was measured in flat-bottom 96-well plates (Greiner) in 200 μL sample using a Synergy MX microplate reader (BioTek). The light path was calculated as 0.632 cm and used to adjust the OD_600_ values to the standard 1 cm light path. CO_2_ production in white grape juice fermentations was estimated by measuring the weight of the bottles with the closed waterlock before and after the fermentation. The difference was assumed to be due to loss of CO_2_ (van Rijswijck et al., 2017).

Sugars were measured by High Pressure Liquid Chromatography (HPLC) on an ICS5000 HPLC system (Thermo Scientific) equipped with a DP pump, AS-AP autosampler and a VWD UV detector (Dionex), at 210 nm and an RI detector (Shodex) at 35°C. An HPX-87H cation-exchange column (Aminex) was used with a mobile phase of 0.016 N H_2_SO_4_. The HPLC was operated at 0.8 mL/min and 60°C. A final concentration of 2 mM DMSO in 0.04 N H_2_SO_4_ was used as internal standard.

Ethanol was measured by GC-FID as described before (Kruis et al., 2017) on a Shimadzu 2010 gas chromatograph equipped with a temperature controlled 20i-s autosampler. 0.5 μL of liquid sample was injected on a Stabilwax column (30 m × 0.25 mm, 0.5 μm coating, Restek). The column temperature was held at 60°C for 1 min and increased to 120°C at a rate of 20°C/min. The split ratio was 20. 2 mM 1-butanol was used as internal standard.

The ester production profile of the yeast strains was assessed using Headspace-Solid Phase Microextraction Gas Chromatography-Mass Spectrometry (HS-SPME GC-MS) as described previously, with minor adaptations (van Rijswijck et al., 2017). A Trace 1300 Gas Chromatograph (Thermo Fisher) with a TriPlus RSH autosampler (Thermo Fisher) and an ISQ QD mass spectrometer (Thermo Fisher) was used for analysis of volatiles. Frozen samples were incubated at 60°C for 10 min. Volatile compounds were extracted for 20 min at 60°C using an SPME fiber (Car/DVB/PDMS, Supelco). The compounds were desorbed from the fiber for 2 min onto a Stabilwax^®^–DA column (30 m length, 0.25 mm ID, 0.5 μm d_f_, Restek). The PTV was heated to 250°C and operated in split mode at a ratio of 1:25. The GC oven temperature was kept at 40°C for 2 min, raised to 240°C with a slope of 10°C/min and kept at 240°C for 5 min. Helium was used as carrier gas at a constant flow rate of 1.2 ml/min. Mass spectral data were collected over a range of m/z 33–250 in full-scan mode with 3.0030 scans/s. Data was analyzed using Chromeleon^®^ 7.2. The ICIS algorithm was used for peak integration and the NIST main library to match the mass spectral profiles with the profiles of NIST. Peak areas were calculated using the MS quantification peak (highest m/z peak per compound).

### Statistical Analysis

Statistical analysis was performed using R (R Foundation for Statistical Computing, 2016). A one tailed Student’s *t*-test was performed. The *p*(critical) values were adjusted according to the Bonferroni method (Dunn, 1961) to account for multiple comparisons.

### Bioinformatics

The protein-coding nucleotide sequences of *ATF1, ATF2, EAT1, EEB1, EHT1*, and *IMO32* were retrieved from the 157 *S. cerevisiae* strains sequenced in Gallone et al. (2016) (*de novo* assemblies downloaded from NCBI BioProject accession: PRJNA323691). Local BLAST databases were set up for all the genomes and BLASTN (vBLAST+ 2.5.0) searches were performed (1E-04 *E*-value cut-off). The protein coding sequences of each gene from *S. cerevisiae* CEN.PK2-1D were used as queries. Multiple sequence alignments (MSAs) were obtained for each gene using MAFFT (v7.187), with default settings and 1,000 refinement iterations (Katoh and Standley, 2013). Maximum likelihood (ML) trees were constructed for each MSA using RAxML (v8.2.8), under the GTRGAMMA model (Stamatakis, 2014). Rapid bootstrapping (2000 bootstrap replicates) and search for the best-scoring ML tree were conducted in one single run (Pattengale et al., 2009). Trees were visualised and rooted in FigTree (v1.4.2) using *Saccharomyces paradoxus* (Scannell et al., 2011) as outgroup^1^. Shannon entropy (H) was calculated for every position within the protein translated alignment of each gene (Shannon, 1948). Gaps were excluded from the calculation. Estimated ploidy and copy number variation data were obtained from Gallone et al. (2016).

## Results

### Genomic Analysis of AATs in Industrial *S. cerevisiae* Strains

**FIGURE 1:**
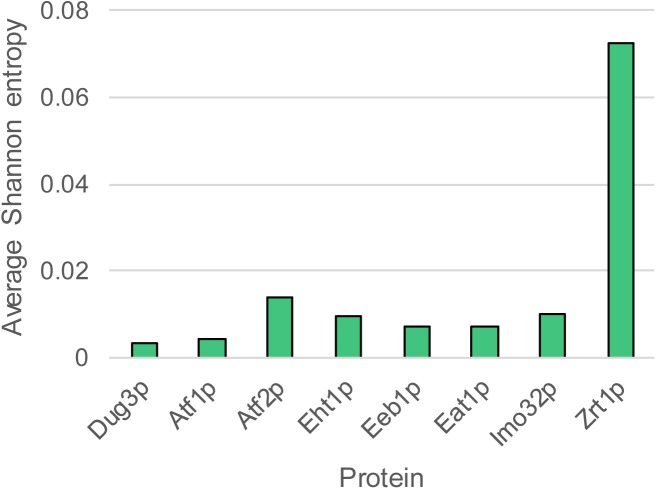
Conservation of the six AAT protein sequences. The average Shannon entropy was calculated as the mean of Shannon entropies at individual amino acid positions. Higher Shannon entropy indicates higher conservation.

Ester production profiles of diverse *S. cerevisiae* strains used in beer brewing, wine making and other industrial processes vary significantly. Gallone et al. (2016) previously reported the detection of ethyl acetate, isoamyl acetate, ethyl hexanoate and ethyl octanoate production by 157 industrial yeast strains with known genome sequences. The total amount of esters produced by the strains varied from 7 to 57 mg/L. Ethyl acetate was the most abundant ester (88.0–98.5% of the esters measured), followed by isoamyl acetate (0.9–10.0% of the esters measured). Ethyl hexanoate and ethyl octanoate constituted between 0.1 and 3.0% of the total ester produced by the strain collection. We investigated whether this large variability can be explained by differences at the genomic level. We compared the sequence diversity and copy number variation (CNV) of *ATF1, ATF2, EHT1, EEB1, IMO32*, and *EAT1*. All six genes were present in all strains of the yeast collection. The conservation of the translated nucleotide sequences was measured using Shannon entropy (Shannon, 1948). This method calculates the variability of each amino acid position in the sequence alignment. The conserved DUG3 and the variable ZRT1 genes were included in the analysis as a control. The average Shannon entropy of the protein sequences showed that Atf2p was the most variable while Atf1p was the most conserved AAT (Figure 1). However, all AATs were generally highly conserved, relative to DUP3 and ZRT1. We sorted the ester production profiles of the yeasts according to the phylogenetic distribution of each individual AAT. Even upon visual inspection it became clear that there was little to no correlation between the primary protein-sequence variation of the AATs and the ester production profiles (Supplementary Figure S1).

A typical characteristic of industrial *S. cerevisiae* strains is their high variability in chromosome content (ploidy). These strains often exhibit abnormal numbers of chromosomes relative to their expected ploidy (aneuploidy), or several small and local changes in CNV, such as duplications and deletions (Gallone et al., 2016). Based on the estimated ploidy levels and copy number profiles reported by Gallone et al. (2016), we examined the copy number levels of each AAT gene. Generally, copy number changes of the AAT genes were associated with duplications and deletions involving full chromosomes or large chromosomal fragments, relative to the rest of the genome. The comparison of the copy number changes of each AAT with their ester production profiles again showed no correlation with either the total ester amount produced, or with the ratios between the four measured esters (data not shown). The results of the *in silico* analysis suggest that the dramatic differences in ester production in the yeast collection cannot be explained by the variation of the coding regions of the AATs on a genomic level.

### Overexpression of the Eat Family Increases Acetate and Propanoate Ester Production

**FIGURE 2:**
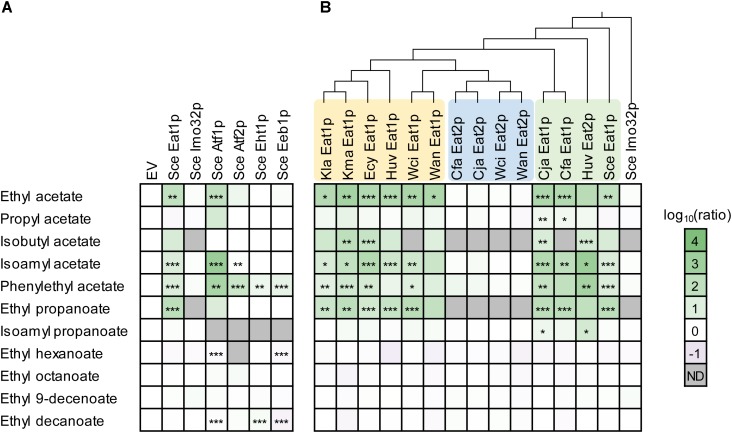
Overexpression of yeast AATs in *Saccharomyces cerevisiae* CEN.PK2-1D. **(A)** Overexpression of the known *S. cerevisiae* AATs. **(B)** Ester production profiles of *S. cerevisiae* CEN.PK2-1D expressing 15 *eat* homologs from nine yeast species. Each square represents the log_10_ of the ratio between the ester peak area of an overexpression strain relative to the area detected in the empty vector (EV) strain. Values represent the average of two biological replicates. Stars indicate statistical significance: ^∗^α = 0.1, ^∗∗^α = 0.05, ^∗∗∗^α = 0.01. The *p*(critical) values after the Bonferroni adjustment were 0.0067, 0.0033, and 0.0007, respectively. Wan, *Wickerhamomyces anomalus*; Wci, *Wickerhamomyces ciferrii*; Kma, *Kluyveromyces marxianus*; Kla, *Kluyveromyces lactis*; Cja, *Cyberlindnera jadinii*; Cfa, *Cyberlindnera fabianii*; Huv, *Hanseniaspora uvarum*; Ecy, *Eremothecium cymbalarie;* Sce, *Saccharomyces cerevisiae*; ND, not determined.

We determined the effect of *ATF1, ATF*2, EHT1, EEB1, IMO32, and EAT1 overexpression on ester production in S. cerevisiae CEN.PK2-1D. This haploid yeast strain was previously sequenced (Nijkamp et al., 2012) and contains only one copy of each AAT gene. The parental strain produced five different acetate esters (ethyl-, propyl-, isobutyl-, isoamyl-, and phenylethyl acetate), and two propanoate esters (ethyl- and isoamyl propanoate). These esters were produced from acetyl-CoA or propionyl-CoA, and a range of (fusel) alcohols formed innately by *S. cerevisiae* CEN.PK2-1D. The strain also produced four ethyl-MCFA esters (ethyl -hexanoate, -octanoate, -decenoate, and -decanoate). Overexpression of *ATF1* resulted in a broad increase in acetate ester production, while overexpression of *ATF2* caused increased levels of phenylethyl acetate and to a lesser extent isoamyl acetate production. An increase in phenylethyl acetate levels was also observed in strains overexpressing *EHT1* and *EEB1*, although this increase was much lower compared to *ATF1* and *ATF2* (Figure 2A). The strain overexpressing *EAT1* produced more acetate esters and ethyl propanoate but had no effect on MCFA production. The overexpression of *IMO32* had no effect on ester production (Figure 2A).

Next, we assessed whether acetate and propanoate ester production were increased by other *EAT* genes. We overexpressed 15 members of the *EAT* family in *S. cerevisiae* CEN.PK2-1D, originating from nine yeast species (Figure 2B). It was found that 10 out of 15 overexpression strains showed increased production of acetate and propanoate esters, while medium chain fatty acid (MCFA) ester production was not affected. This was comparable to the overexpression of the *S. cerevisiae EAT1*. Ordering the ester production profiles of the overexpression strains according to a phylogenetic tree of the Eat protein sequences revealed that three groups seem to exist within the Eat family (Figure 2B). Two groups consist of *EAT1* genes that are able to increase ester production. The third group consists of genes which had only a negligible effect on the ester profile compared to the other two *EAT* groups. It is possible that these enzymes are not involved in ester synthesis in yeast, although it cannot be excluded that these genes are not expressed well in *S. cerevisiae* CEN.PK2-1D. Curiously, *W. anomalus* and *W. ciferrii EAT1* are more closely related to the non-producing *EAT* genes, but still increased ester production in *S. cerevisiae* CEN.PK2-1D. *S. cerevisiae* CEN.PK2-1D (pCUP1:Huv2) showed a different ester production profile compared to the other *EAT1* expressing strains. The strain showed a 1291- and 590-fold increase in isoamyl acetate and phenylethyl acetate production, respectively (Supplementary Figure S2). These were some of the highest increases in ester production observed in this experiment. Huv *EAT2* was also the only gene that significantly (>5-fold) increased the production of isoamyl propanoate. The remaining *EAT1* homologs evoked comparable ester production profiles to *S. cerevisiae* CEN.PK2-1D (pCUP1: Sce Eat1).

### Effect of EAT Disruption on *in vivo* Ester Production in *S. cerevisiae*

*Saccharomyces cerevisiae EAT1* clearly has the potential to produce acetate and propanoate esters *in vivo*, while the effect of *IMO32* is unconfirmed. To determine the impact of *EAT1* and *IMO32* on the total ester production, we disrupted the two genes in combination with the other four known *S. cerevisiae* AAT genes (*ATF1, ATF2, EHT1*, and *EEB1*) in *S. cerevisiae* CEN.PK2-1D by CRISPR-Cas9 (DiCarlo et al., 2013; Mans et al., 2015). A total of 14 disruption strains were generated; six single knockouts (Δ*atf1*,Δ*atf2*,Δ*eht1*,Δ*eeb1*,Δ*eat1*,Δ*imo32*), two double knockouts (Δ*atf1*Δ*atf2*,Δ*eht1*Δ*eeb1*), two triple knockouts (Δ*atf1*Δ*atf2*Δ*eat1*,Δ*eht1*Δ*eeb1*Δ*eat1*), one quadruple knockout (Δ*atf1*Δ*atf2*Δ*eht1*Δ*eeb1*), two quintuple knockouts (Δ*atf1*Δ*atf2*Δ*eht1*Δ*eeb1*Δ*eat1*, Δ*atf1*Δ*atf2*Δ*eht1*Δ*eeb1*Δ*imo32*), and finally a sextuple disruption strain in which all six genes were disrupted (Δ*atf1*Δ*atf2*Δ*eht1*Δ*eeb1*Δ*eat1*Δ*imo32*). We determined their ester production profiles under three cultivation conditions. Two were based on routine laboratory cultivations in minimal YSg medium and rich YPD-80 medium while shaking. The latter medium was used previously to assess the effects of AAT disruptions on ester production (Verstrepen et al., 2003b). The third cultivation condition simulated industrial white wine fermentations and was performed statically. We first assessed the fermentation performance of the strains and found that there were only small differences in growth, glucose consumption or ethanol production in either YSg medium or YPD-80 medium (Supplementary Figure S3). However, the strains showed substantial differences in their ester production profiles. Three acetate esters (ethyl-, isoamyl-, and phenylethyl acetate) and three ethyl MCFA esters (ethyl- octanoate, 9-decenoate, and decanoate) were measured under these cultivation conditions.

**FIGURE 3:**
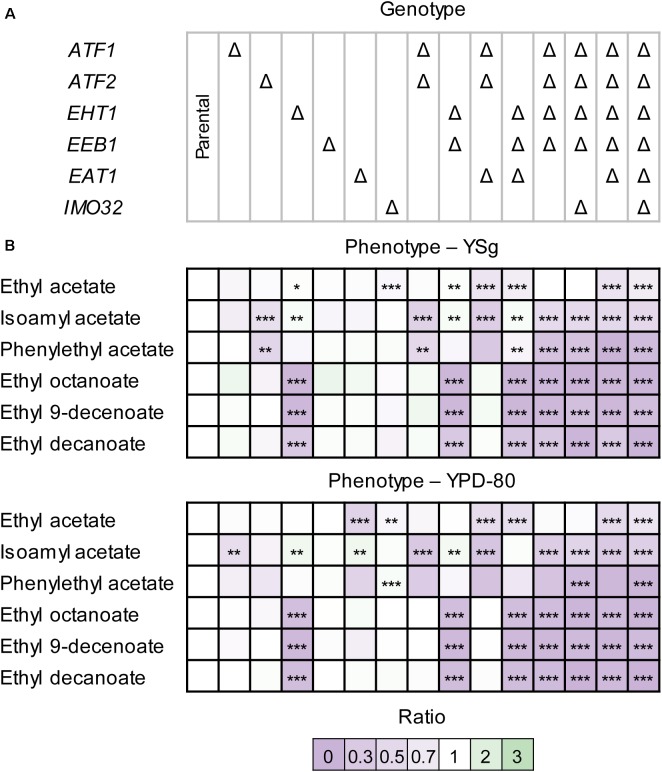
Ester production profiles of *S. cerevisiae* CEN.PK2-1D AAT disruption strains. **(A)** Strains grown on YSg (minimal) medium. **(B)** Strains grown on YPD-80 (rich medium). Strains were cultivated in 10 mL medium while shaking. Ester production was measured by HS-SPME GC-MS. Values represent the ratio between the area of the ester peak relative to the peak measured in the parental strain. The values are averages of two technical and two biological replicates. Values represent the average of two biological replicates. Stars indicate statistical significance: ^∗^α = 0.1, ^∗∗^α = 0.05, ^∗∗∗^α = 0.01. The *p*(critical) values after the Bonferroni adjustment were 0.0067, 0.0033, and 0.0007, respectively.

Single deletions of *ATF1* and *ATF2* resulted in a slightly lower acetate ester production in both YSg (Figure 3A) and YPD-80 medium (Figure 3B). In contrast, CEN.PK2-1D Δ*eht1* produced less MFCA esters, but showed no differences in acetate ester levels in either media (Figure 3). The effects of these single disruptions were observed previously (Verstrepen et al., 2003b; Saerens et al., 2006), although the effect of the *ATF1* and *ATF2* deletions on acetate ester production in these studies was stronger. This may be due to the repression of *ATF1* under aerobic conditions (Mason and Dufour, 2000). Disruption of *EEB1* did not show any detectable effect on ester production in YSg or YPD-80 medium (Figure 3). CEN.PK2-1D Δ*eat1* showed significantly reduced levels of ethyl acetate in YPD-80 medium (Figure 3B). In some cases, the roles of the AAT genes in ester production became more apparent when multiple genes were disrupted simultaneously. For example, the ethyl acetate production of CEN.PK2-1D Δ*eat1* grown on YSg medium was similar to the CEN.PK2-1D parental strain. However, ethyl acetate production was reduced in CEN.PK2-1D Δ*atf1*Δ*atf2*Δ*eat1*, CEN.PK2-1D Δ*eht1*Δ*eeb1*Δ*eat1* and CEN.PK2-1D Δ*atf1*Δ*atf2*Δ*eht1*Δ*eeb1*Δ*eat1* compared to their respective strains without the *eat1* deletion (*p* < 0.0007). The gene disruptions mostly showed a cumulative effect. The quintuple disruption strain CEN.PK2-1D Δ*atf1*Δ*atf2*Δ*eht1*Δ*eeb1*Δ*eat1* produced the least esters. However, ester production was not completely abolished, indicating that other ester forming reactions exist in *S. cerevisiae* CEN.PK2-1D. This was the most apparent in the case of ethyl acetate and isoamyl acetate, where more than 50% ethyl acetate production remained in some cases (Supplementary Figure S4). Imo32p was previously associated with ester production in the absence of Atf1p (Holt et al., 2018). The single deletion strain CEN.PK2-1D Δ*imo32* produced 10% less ethyl acetate in both YSg and YPD-80 media. This effect disappeared when *IMO32* was disrupted in CEN.PK2-1D Δ*atf1*Δ*atf2*Δ*eht1*Δ*eeb1* and CEN.PK2-1D Δ*atf1*Δ*atf2*Δ*eht1*Δ*eeb1*Δ*eat1* (Figure 3). The strain where all six genes were disrupted still produced similar amounts of esters compared to CEN.PK2-1D Δ*atf1*Δ*atf2*Δ*eht1*Δ*eeb1*Δ*eat1*. This indicates that *IMO32* does not play a direct role in ester production in *S. cerevisiae* CEN.PK2-1D grown on YSg and YPD-80 media. The production of other esters, such as phenylethyl acetate and all MCFA esters was almost completely eliminated in CEN.PK2-1D Δ*atf1*Δ*atf2*Δ*eht1*Δ*eeb1*Δ*eat1* and CEN.PK2-1D Δ*atf1*Δ*atf2*Δ*eht1*Δ*eeb1*Δ*eat1*Δ*imo32* grown on YSg or YPD-80 medium (Supplementary Figure S4). The effect of the AAT disruptions was then investigated under more industrially relevant conditions. The strains were cultivated statically in white grape juice, which contained 112.1 g/L sugars (58.8 g/L glucose and 53.3 g/L fructose). The gene disruptions affected the fermentation performance of the strains. This was most apparent in the strain where all AAT genes were disrupted. The growth, sugar consumption, ethanol formation and CO_2_ formation were approximately 10% lower (Supplementary Figure S5). Five additional esters (isobutyl acetate, ethyl hex-4-enoate, ethyl hexanoate, isoamyl octanoate, and ethyl dodecanoate) were detected in white grape juice compared to YSg and YPD-80 medium (Figure 3).

**FIGURE 4:**
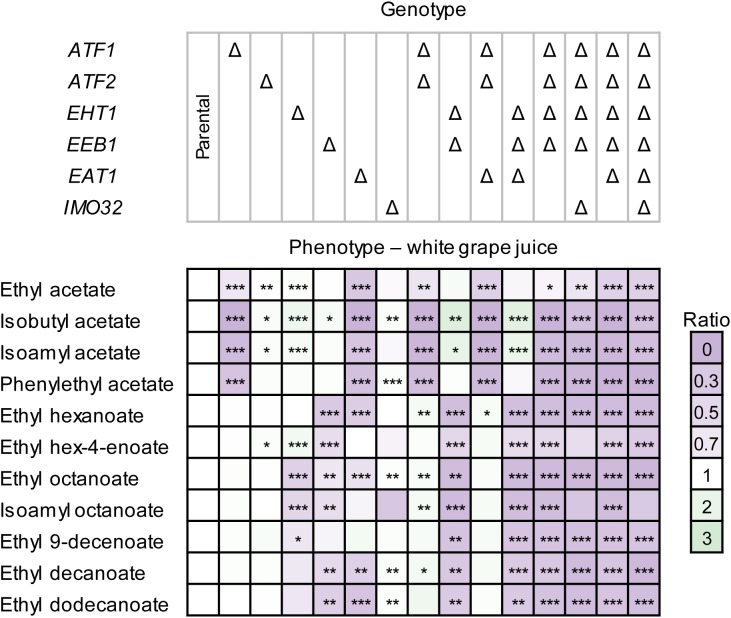
Ester production profiles of *S. cerevisiae* CEN.PK2-1D AAT disruption strains in white grape juice. Strains were cultivated statically in 50 mL medium for 7 days. Ester production was measured by HS-SPME GC-MS. Values represent the ratio between the area of the ester peak relative to the peak measured in the parental strain. The values are averages of two technical and two biological replicates. Stars indicate statistical significance: ^∗^α = 0.1, ^∗∗^α = 0.05, ^∗∗∗^α = 0.01. The *p*(critical) values after the Bonferroni adjustment were 0.0067, 0.0033, and 0.0007, respectively.

The AAT disruption strains produced less esters in white grape juice compared to the parental strain (Figure 4). The lower production could hypothetically be explained by the reduced fermentation performance of the disruption strains. However, the changes in ester production were more dramatic than, e.g., the reduction of growth or sugar consumption. For example, CEN.PK2-1D Δ*atf1*Δ*atf2*Δ*eht1*Δ*eeb1*Δ*eat1* consumed approximately 10% less sugar than the parental strain, while ester production was decreased by more than 90% (Figure 4). The independence of ester formation from growth in this experiment was also apparent by comparing the relevant disruption strains. For example, CEN.PK2-1D Δ*atf1*Δ*atf2*Δ*eht1*Δ*eeb1* and CEN.PK2-1D Δ*atf1*Δ*atf2*Δ*eht1*Δ*eeb1*Δ*eat1* showed no significant differences in fermentation performance (Supplementary Figure S5), whereas ethyl acetate and phenylethyl acetate were almost 50% or more lower in the strain where *EAT1* was disrupted (Supplementary Figure S6, *p*-value < 0.0007). This result supports the observation in YSg and YPD-80 media (Figure 3).

Some CEN.PK2-1D AAT disruption strains behaved differently in white grape juice (Figure 4) compared to the cultivation in YSg and YPD-80 media. The most notable difference was observed in CEN.PK2-1D Δ*eat1*, which showed a decreased production of both acetate and MCFA esters. This observation is not in line with the behavior of CEN.PK2-1D Δ*eat1* in laboratory media (Figure 3), or the overexpression experiment (Figure 3), where only an effect on acetate and propanoate ester production was observed. The deletion of *EAT1* did not show the same effect on MCFA ester production in CEN.PK2-1D Δ*atf1*Δ*atf2*Δ*eat1*, compared to CEN.PK2-1D Δ*atf1*Δ*atf2*. Rigorous genotyping and three independent repeats of the fermentation confirmed that the observed results are reproducible and not caused by technical issues. Moreover, CEN.PK2-1D Δ*eat1* served as the precursor strain for generating other disruption strains that did not show this behavior.

Disruption of *ATF2* and *EEB1* also resulted in different production profiles in white grape juice, compared to YSg and YPD-80. CEN.PK2-1D Δ*atf2* did not show a difference in the ester production profile compared to the parental strain, whereas it reduced acetate ester production in YSg and YPD-80 cultures. In contrast, the disruption of *EEB1*, which had no significant effect in laboratory media, resulted in a broad decrease of MCFA esters in white grape juice.

Deletion of *ATF1* and *EHT1* showed reduced production of acetate and MCFA esters, respectively, which is comparable to the profiles observed in laboratory media (Figure 3). The quintuple disruption strain in CEN.PK2-1D Δ*atf1*Δ*atf2*Δ*eht1*Δ*eeb1*Δ*eat1* again produced the least esters. The disruption of *IMO32* either did not have an impact on ester production in wine juice, or these differences were not statistically significant (Figure 4). Residual levels of esters were still detected (Supplementary Figure S6), matching the observations in YSg and YPD-80 media (Figure 3).

## Discussion

Ester production by *S. cerevisiae* is critical for the flavor and aroma of fermented foods. Industrial yeasts strains can vary dramatically in both the amounts of esters produced, as well as the ratios between the different esters (Gallone et al., 2016). We attempted to correlate the differences in ester production to variations of the amino acid sequences and CNV of known *S. cerevisiae* AATs. We observed no clear correlation, indicating that other factors determine the differences in ester production. These may include factors such as different expression levels of the AAT genes or varying levels of esterase activity (Verstrepen et al., 2003b; Lilly et al., 2006). The role of Eat1p on ester production was instead determined *in vivo* in *S. cerevisiae* CEN.PK2-1D.

Strains in which *EAT1* was deleted exhibited significantly lower acetate ester levels, while overexpression of the gene increased the production of acetate as well as propanoate esters. This shows that Eat1p clearly contributes to the *in vivo* synthesis of these esters. The Eat family seems to prefer acetyl-CoA and propionyl-CoA as the acyl-donor. However, the *S. cerevisiae* Eat1p is located in the mitochondria (Huh et al., 2003) where mostly acetyl-CoA is produced. On the other hand, MCFA acyl-CoA synthesis predominantly takes place in the cytosol (Marchesini and Poirier, 2003; Tehlivets et al., 2007). It is therefore possible that Eat1p might be able to use other acyl-CoAs, but they are inaccessible under *in vivo* conditions. Contrary to acyl-CoA, alcohols can freely diffuse throughout the cell, and may react with the acyl-donor anywhere in the cell. This may explain why Eat1p seems to be unspecific toward alcohols but prefers short chain acyl-CoAs. Curiously, a study where the *S. cerevisiae EAT1* was overexpressed found that Eat1p was mostly limited to the production of ethyl acetate (Holt et al., 2018). The substrate preferences of the enzymes should be determined *in vitro*. Under *in vivo* conditions, the Eat family produces a similar spectrum of esters as Atf1p, although ethyl propanoate (pineapple aroma) was increased significantly more by Eat1p. There is variation in the individual ester levels produced by the Eat family as well. For example, expression of the *Hanseniaspora uvarum* Eat2 in *S. cerevisiae* had a bigger impact on isoamyl acetate and phenylethyl acetate production compared to ethyl acetate. In many cases, a high ratio of isoamyl acetate over ethyl acetate results in more pleasant aromas of fermented products (Verstrepen et al., 2003a).

Atf1p, Atf2p, Eht1p, Eeb1p, and Eat1p are not the only enzymes contributing to ester synthesis in *S. cerevisiae*. The production of some esters was almost completely abolished in the quintuple disruption strain CEN.PK2-1D Δ*atf1*Δ*atf2*Δ*eht1*Δ*eeb1*Δ*eat1*, while some were still produced at reduced levels compared to the parental strain. Esters that were almost completely abolished include isobutyl acetate, phenylethyl acetate and ethyl propanoate and most MCFA esters. On the other hand, there were still considerable levels of ethyl acetate and isoamyl acetate produced even when all five AAT genes were disrupted. This is somewhat surprising since previous reports showed that ethyl acetate production was reduced by 50% when either *ATF1* or *EAT1* were disrupted (Verstrepen et al., 2003b; Kruis et al., 2017). The CEN.PK2-1D Δ*atf1*Δ*atf2*Δ*eat1* strain was therefore not expected to produce ethyl acetate anymore. The additional disruption of *IMO32* did not explain this residual ester production. It is clear that there are other mechanisms producing ethyl acetate and isoamyl acetate in *S. cerevisiae*. It is possible that the esters are synthesized by other unknown AATs or through entirely different enzymatic reactions. In *S. cerevisiae*, ester synthesis has also been linked to hemiacetal dehydrogenation and reverse esterase activity (Kusano et al., 1998; Park et al., 2009).

The three cultivation conditions strongly influenced the effects of the AAT disruptions on strain ester profiles. In CEN.PK2-1D Δ*eat1* grown in YPD-80 or white grape juice produced less esters even as a single disruption. In white grape juice the single deletion of *EAT1* even reduced MCFA ester production. This effect was unexpected since it was not observed in any other strain where *EAT1* was disrupted. The three independent repetitions of the experiment consistently showed the same result. Atf2p and Eeb1p also behaved differently in the three media. The deletion of *ATF2* showed decreased acetate ester production when grown in YSg and YPD-80 while shaking, but not in the static white grape juice fermentations. Strains where *EEB1* was disrupted showed the opposite trend. It is possible that the effect of the *ATF2*, *EEB1* and other AAT genes is controlled by the presence of oxygen. In several cases, strain genotype also influenced the effect individual AAT disruptions had on the total ester profile. For example, in YSg medium, the effect of *EAT1* disruption was not apparent in CEN.PK2-1D Δ*eat1*. However, when the gene was disrupted in various combinations with the remaining four AATs, it caused a decrease in acetate ester production. This indicates that in some cases, the effect of a single AAT disruption may be masked by the remaining AATs, which is plausible as they compete for the same substrates; although not all AATs have the same cellular location (Huh et al., 2003; Lin and Wheeldon, 2014; Holt et al., 2018). There is a significant knowledge gap on the expression, function and interaction of yeast AATs, especially under industrially relevant conditions. Further research into these aspects might reveal the reasons behind the unexpected behavior of some of the disruption strains. Nevertheless, the general trend observed both in the overexpression, as well as the deletion strains is that Eat1p is a major source of acetate and propanoate esters and is able to utilize a wide range of alcohols.

The esters produced by Eat1p and the other AATs present in yeast are key flavor compounds in fermented products. We have shown that Eat1p contributes to the synthesis of acetate and propanoate esters in *S. cerevisiae*. However, we have also demonstrated that even when all known AATs were disrupted, ester synthesis was not completely abolished. This study provided a better understanding of ester production in yeast but has also focused our attention to the still existing knowledge gap on ester formation by *S. cerevisiae*.

## Author Contributions

AK, BG, IvR, ES, and RW conceived and designed the work. AK and TJ performed the wet-lab experiments. BG performed the *in silico* genomic analysis. AK and BG wrote the manuscript. AM, IvR, JW–R, JS, ES, KV, SK, JvdO, and RW critically read and revised the manuscript. All authors read and agreed to the submission of the manuscript.

## Conflict of Interest Statement

The authors declare that the research was conducted in the absence of any commercial or financial relationships that could be construed as a potential conflict of interest.

## Footnotes

1 http://tree.bio.ed.ac.uk/software/figtree/
