# Supplementary material for: Contribution of Eat1 and Other Alcohol Acyltransferases to Ester Production in Saccharomyces cerevisiae
Source: Front Microbiol. 2018 Dec 21;9:3202. doi: 10.3389/fmicb.2018.03202 (PMC6308380; doi:10.3389/fmicb.2018.03202)
Supplement: Supplementary file 1 [file Data_Sheet_1.PDF]

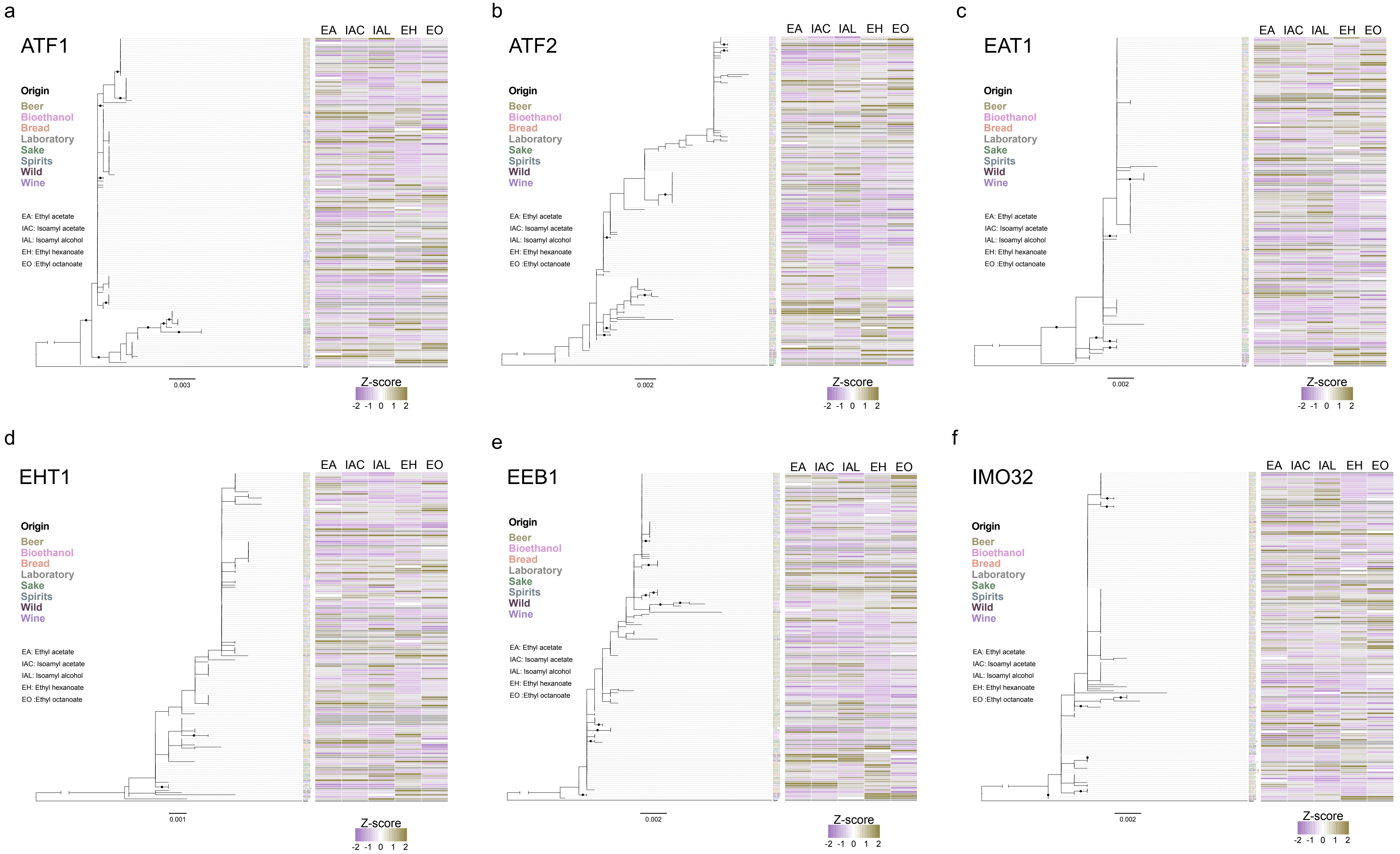

Supplementary Figure 1: Ester production profiles in a collection of industrial Saccharomyces cerevisiae strains (Gallone et al., 2016) sorted according to the best phylogenetic hypothesis of Atf1, Atf2, Eht1, Eeb1, Eat1 and Imo32 protein coding sequences (panels a, b, c, d, e ,f, respectively).
Colour codes indicate origin of the strains. Branches supported by more than 60% of 2000 bootstrap replicates are highlighted by black dots.
