# Supplementary material for: Contribution of Eat1 and Other Alcohol Acyltransferases to Ester Production in Saccharomyces cerevisiae
Source: Front Microbiol. 2018 Dec 21;9:3202. doi: 10.3389/fmicb.2018.03202 (PMC6308380; doi:10.3389/fmicb.2018.03202)
Supplement: Supplementary file 2 [file Data_Sheet_2.docx]

Supplementary Figure 2: Overexpression of 15 *eat1* homologs from nine yeast species in *S. cerevisiae* CEN.PK2-1D. The colours of the squares represent the log ratio between the ester peak area of an overexpression strain relative to the area detected in the empty vector (EV) strain. The numbers in the squares represents the ratio between the ester peak area of an overexpression strain relative to the area detected in the empty vector (EV) strain. The values are averages of two biological replicates. The Eat1 homologs form three phylogenetic groups. Two groups (yellow and green) increased ester production while one (blue) had no effect on ester production. Abbreviations: Wan - *Wickerhamomyces anomalus*, Wci - *Wickerhamomyces ciferrii*, Kma - *Kluyveromyces marxianus*, Kla - *Kluyveromyces lactis*, Cja - *Cyberlindnera jadinii*, Cfa - *Cyberlindnera fabianii*, Huv - *Hanseniaspora uvarum*, Ecy - *Eremothecium cymbalarie,* Sce - *Saccharomyces cerevisiae*

Supplementary Figure 3: Fermentation profile of *S. cerevisiae* CEN.PK2-1D AAT disruption strains. **(A)** – strains grown on YSg (minimal) medium. **(B)** – strains grown on YPD-80 (rich medium). Strains were cultivated in 10 mL medium while shaking. Sugars and ethanol were measured by HPLC and GC, respectively after 20 hours of cultivation. The values shown are averages of two biological duplicates. Error bars represent the standard deviation.

Supplementary Figure 4: Ester production profiles of *S. cerevisiae* CEN.PK2-1D AAT disruption strains. A – strains grown on YSg (minimal) medium. B – strains grown on YPD-80 (rich medium). Strains were cultivated in 10 mL medium while shaking. Ester production was measured by HS-SPME GC-MS. The numbers in the squares represent the ratio between the area of the ester peak relative to the peak measured in the parental strain. The values are averages of two technical and two biological replicates.

Supplementary Figure 5: Fermentation profile of *S. cerevisiae* CEN.PK2-1D AAT disruption strains in white grape juice. Strains were cultivated statically in 50 mL medium for 7 days. The medium initially contained 112.1 g/L fermentable sugars. Sugars and ethanol were measured by HPLC and GC, respectively. CO_2_ production was measured as the loss of weight before and after the fermentation. The values shown are averages of two biological duplicates. Error bars represent the standard deviation.

Supplementary Figure 6. Ester production profiles of *S. cerevisiae* CEN.PK2-1D AAT disruption strains in white grape juice. Strains were cultivated statically in 50 mL medium for 7 days. Ester production was measured by HS-SPME GC-MS. The numbers in the squares represent the ratio between the area of the ester peak relative to the peak measured in the parental strain. The values are averages of two technical and two biological replicates.
